# The stress-activated kinase p38 mediates non-canonical activation of Src and tyrosine phosphorylation of the adapter protein TAB1

**DOI:** 10.1016/j.jbc.2026.111200

**Published:** 2026-01-23

**Authors:** Iimi Onuma, Yusuke Iwata, Yue Zhou, Mai Nakada, Arisa Kondo, Hiroyuki Iwahara, Kanako Natori, Satoru Yokoyama, Kazuyasu Chihara, Kenji Takeuchi, Kiyonao Sada, Tatsuhiko Ozawa, Mineyuki Mizuguchi, Nobuyuki Yamagishi, Michael Kracht, Hiroaki Sakurai

**Affiliations:** 1 Department of Cancer Cell Biology, Faculty of Pharmaceutical Sciences, University of Toyama, Toyama, Japan; 2 Department of Genome Science and Microbiology, Faculty of Medical Sciences, University of Fukui, Fukui, Japan; 3 Department of Immunology, Faculty of Medicine, Academic Assembly, University of Toyama, Toyama, Japan; 4 Center for Advanced Antibody Drug Development, University of Toyama, Toyama, Japan; 5 Faculty of Pharmaceutical Sciences, University of Toyama, Toyama, Japan; 6 Laboratory of Analytics for Biomolecules, Faculty of Pharmaceutical Science, Setsunan University, Osaka, Japan; 7 Rudolf Buchheim Institute of Pharmacology, Justus Liebig University, Giessen, Germany

**Keywords:** TAB1, src, p38, TAK1, FAK, tyrosine kinase

## Abstract

Src is a non-receptor tyrosine kinase that is overexpressed and highly activated in many cancers and is one of the key factors contributing to malignant transformation. According to current concepts, Src activity relies on tyrosine phosphorylation, and phospho(p) Y419 in the activation loop is often regarded as a marker of its activation. However, recent studies have shown that pY419 may contribute to substrate selection. Therefore, the mechanisms underlying Src activation other than classical tyrosine phosphorylation warrant further study. We herein demonstrated that Src phosphorylates a novel substrate, TAB1, directly at Y481 in the TAK1-binding domain, changing the TAK1-TAB1 interaction. p38 enhances Src-mediated TAB1 phosphorylation through the direct phosphorylation of Src at N-terminal S75. Moreover, the mode of substrate recognition by the SH2 domain of Src is different in TAB1 than in FAK, a known SH2-dependent substrate. The present results identify a novel non-canonical Src activation mechanism based on serine phosphorylation and suggest pY481-TAB1 and pS75-Src as improved markers of Src activation, thereby offering alternative modes for assessing the Src activation status of Src-dependent cancers before and during targeted therapy.

*v-Src* encoded by the Rous sarcoma virus was the first proto-oncogene to be discovered (1, 2, 3, 4). According to the TCGA database, more than 30% of human cancers harbor *Src* copy number gain, although there are a few activating mutations (5, 6). Src protein expression and activity increase during tumorigenesis and malignant progression (7, 8, 9), which has prompted multiple and ongoing investigations on the mechanisms underlying the activation of Src in cancer.

The kinase activity of Src is mainly regulated by tyrosine phosphorylation and intra-molecular interactions between functional domains. When Y530 in the C-terminal tail is phosphorylated by C-terminal Src kinase (CSK) (10), the SH2 domain intramolecularly captures phosphorylated Y530 (pY530), resulting in a closed inactive conformation (11, 12, 13, 14). The release of the SH2 domain by the dephosphorylation of Y530 and Y419 auto-phosphorylation in the SH1 (catalytic) domain switches the kinase to an active open conformation (15, 16, 17, 18, 19). Some substrate proteins, such as focal adhesion kinase (FAK), bind to the released SH2 domain (20, 21, 22, 23, 24). The carcinogenic v-Src protein is constitutively active (CA) due to the lack of the regulatory C-terminal tail containing Y527 (corresponding to Y530 of human Src). This has led to the concept that the phosphorylation of Y419 in the activation loop is essential for Src activation.

A recent study showed that the Src-Y419F mutant still phosphorylated some substrates, contrary to the conventional mechanism described above, suggesting that Y419 is related to substrate specificity rather than catalytic activity (25). Furthermore, self-autonomous autophosphorylation mechanisms on Y419 and Y530 may promote an allosteric switch between the activation loop and C-terminal tail. High levels of pY419 have been shown to impair the Src-mediated activation of signal transducers and activator of transcription 3 (STAT3) (26, 27). Therefore, the widely accepted concept implicating pY419 in Src activation requires modifications and alternative activation mechanisms need to be considered. The non-canonical phosphorylation of serine/threonine (S/T) residues, such as S75, has been investigated; however, the findings of these analyses have been controversial (28, 29, 30, 31).

Transforming growth factor-β-activated kinase 1 (TAK1) in complex with its activator proteins TAB1 and TAB2 plays a central role in pro-inflammatory cytokine signaling to mitogen-activated kinases (MAPKs) and nuclear factor-κB (32). The C-terminal tail of TAB1 is sufficient to bind to and activate the kinase domain of TAK1 (33, 34). p38-MAPK, a major downstream effector kinase of the TAK1 complex, phosphorylates TAB1 at S/T residues adjacent to the TAK1-binding sequence as a negative feedback mechanism (35, 36). In addition, free TAB1 induces the MAPK kinase (MAP2K)-independent autoactivation of p38α (37), an alternative non-canonical activation mechanism of MAPK, which plays a role in injury during myocardial ischemia (38, 39). Based on these findings, TAB1 is defined as a multifunctional molecule and regulator of TAK1 and p38, and the functional relevance of these mechanisms warrant further study.

In the present study, we identified TAB1-Y481 as a novel Src substrate that is strictly dependent on the phosphorylation status of Src at Y419. We also discovered a p38-mediated Src activation mechanism *via* the direct phosphorylation of Src at S75. The tyrosine phosphorylation of TAB1 provides important insights into non-canonical Src activation mechanisms. Furthermore, the present results revealed a novel crosstalk mechanism between the Src, p38, and TAK1 signaling pathways in cancer.

## Results

### Src directly phosphorylates TAB1-Y481

**Figure 1:**
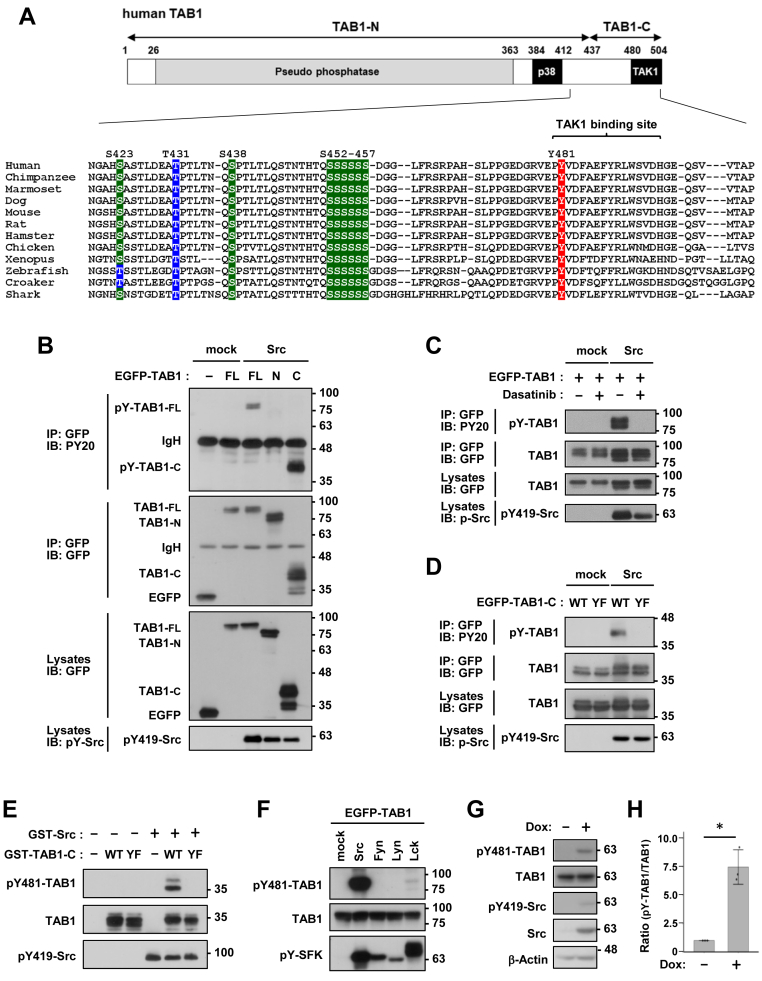
**Src directly phosphorylates TAB1-Y481.***A*, the domain structure of TAB1 and the amino acid sequence around Y481. Conserved phosphorylation serine/threonine and tyrosine sites in the C-terminal regions are highlighted in *green*/*blue* and *red*. *B*, COS-7 cells were transfected with EGFP-tagged full-length (FL), N-terminal (N), or C-terminal (C) TAB1 and Src. *C*, COS-7 cells were transfected with EGFP-TAB1 and Src. Dasatinib (0.1 μM) was added 1 h before cells were harvested. *D*, COS-7 cells were transfected with TAB1-C or its Y481F mutant (YF) and Src. Cell lysates and immunoprecipitates with an anti-GFP antibody were immunoblotted with anti-phosphotyrosine and anti-GFP antibodies. The expression of active Src was detected by an anti-phospho-Src family kinase (Y419) antibody (*B*–*D*). *E*, an *in vitro* kinase assay was performed using recombinant GST-TAB1-C and active GST-Src proteins. *F*, COS-7 cells were transfected with EGFP-TAB1 with SFKs, including Src, Fyn, Lyn, and Lck. *G*, HCT-116-Src cells were treated with 10 ng/ml doxycycline (Dox) for 24 h. TAB1-Y481 phosphorylation and other proteins were detected by an anti-phospho-Y481-TAB1 antibody and the antibodies described above, respectively (*E*–*G*). *H*, the relative quantification of pY481-TAB1, normalized to total TAB1, is presented as the mean ± SD from three independent experiments. *p* values were calculated using Welch’s two-tailed *t* test. ∗*p* < 0.05.

TAB1 has a pseudo-phosphatase domain of unknown function covering most of its N-terminal region as well as the following major functional domains in its C-terminal region (Fig. 1*A*). The TAK1 activation domain is located on the C-terminal tail, of which residues 480 to 495 are sufficient for direct binding to TAK1. The parts adjacent to N-terminal regions are involved in functional interactions with p38; one consists of the S/T cluster (S423/T431/S438 and S452–457) that receives feedback phosphorylation by p38, and the other one is a segment (residues 384–412) that binds directly to and induces the MAP2K-independent auto-activation of p38α (Fig. 1*A*). Based on the results of available phosphoproteomic data (40), we investigated the phosphorylation of highly conserved Y481 in the TAK1-binding domain because the kinase that catalyzes this phosphorylation and its function have not yet been examined.

We initially attempted to identify the kinase for TAB1-Y481. EGFP-tagged TAB1 was transfected with various non-receptor tyrosine kinases in COS-7 cells. The immunoblotting of anti-EGFP immunoprecipitates with an anti-phospho-tyrosine antibody showed that Src selectively induced the tyrosine phosphorylation of TAB1 (Fig. S1). A truncation analysis revealed that the phosphorylated tyrosine residue was in the C-terminal fragment of TAB1 (TAB1-C), but not in TAB1-N, and was blocked by the clinically used Src inhibitor dasatinib (Fig. 1, *B* and *C*). Phosphorylation completely disappeared with the Y481F mutation, suggesting that Y481 was the only residue targeted by Src (Fig. 1*D*). To further confirm this, we conducted an *in vitro* kinase assay using GST-tagged recombinant Src and TAB1-C proteins. Immunoblotting with a newly generated anti-phospho-TAB1-Y481 antibody (41) demonstrated that pY481-TAB1 was detected in wild-type TAB1-C, but not the Y481F mutant, proving the direct phosphorylation of Y481 by Src (Fig. 1*E*). Consistent with the screening results in Fig. S1, the data in Figure 1*F* demonstrate that Src—unlike the other Src family kinases (SFKs) Fyn and Lyn—robustly phosphorylates TAB1. In contrast, another SFK member, Lck, exhibited only minimal phosphorylation activity, markedly weaker than that of Src. Moreover, Src expression by the Tet-on system increased the endogenous level of pY481-TAB1 in HCT-116 colorectal cancer cells (Fig. 1, *G* and *H*). Together, these data establish Src as the most selective and efficient kinase for phosphorylating TAB1 at Y481.

### TAK1 interferes with TAB1 phosphorylation by Src

**Figure 2:**
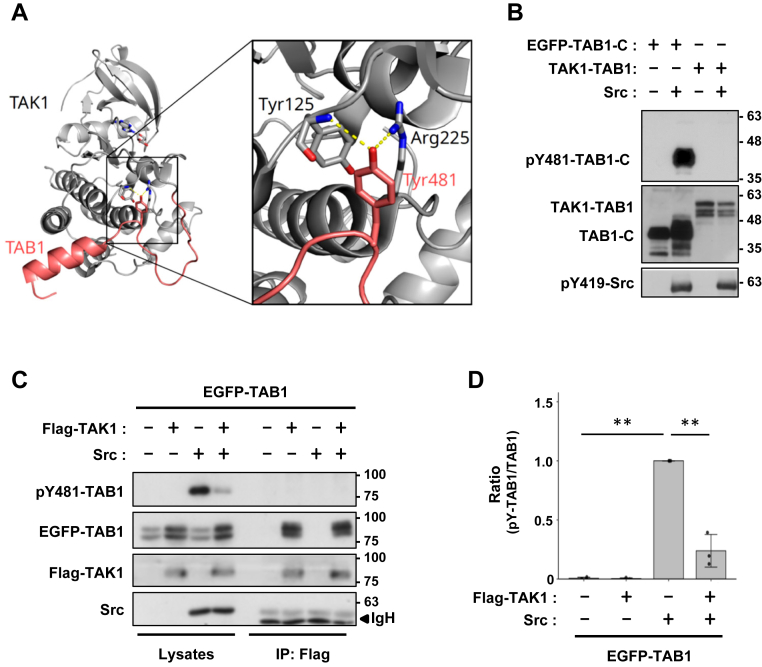
**Src phosphorylates free TAB1 not bound to TAK1.***A*, Crystal structure of the TAK1-TAB1 fusion protein (PDB id: 2EVA). An enlarged version of the interaction interface shows Y125 and R225 of the TAK1 kinase domain forming a hydrogen bond (*yellow dashed line*) with TAB1-Y481. The distance between the backbone amide nitrogen atom of Y125 and the side chain oxygen atom of Y481 is 3.4 Å, while the distance between the guanidinium nitrogen atom (Nη1) of R225 and the side chain oxygen atom of Y481 is 3.1 Å. The figure was generated using PyMol (DeLano Scientific; www.pymol.org). *B*, COS-7 cells were transfected with EGFP-TAB1-C or TAK1-TAB1 with Src. *C*, COS-7 cells were transfected with EGFP-TAB1, Flag-TAK1, and Src. Cell lysates or anti-GFP immunoprecipitates were immunoblotted with primary antibodies against pY481-TAB1, EGFP, pT187-TAK1, TAK1, and pY419-Src. *D*, the relative quantification of pY481-TAB1 in lysates, normalized to total TAB1, is presented as the mean ± SD of three independent experiments. *p* values were calculated by one-way ANOVA followed by either Tukey’s HSD test was applied. ∗∗*p* < 0.01.

Previous crystal structure analyses of a TAK1-TAB1 fusion protein, which consisted of the TAK1 kinase domain linked to the 68 C-terminal amino acids of TAB1 containing the TAK1 activation domain, showed that TAB1-Y481 formed hydrogen bonds on R225 and Y125 of TAK1 within the stable active TAK1-TAB1 conformation (Fig. 2*A*) (42, 43). Therefore, the fusion protein may not have been phosphorylated by Src (Fig. 2*B*). To further investigate the effects of the addition of phosphate to this binding interface, full-length TAK1 was co-transfected with full-length TAB1 and Src. A significant decrease in the level of pY481-TAB1 was observed in the presence of TAK1 (Fig. 2*C*). Furthermore, co-immunoprecipitation revealed that TAB1 bound to TAK1 was not phosphorylated by Src (Fig. 2, *C* and *D*). These results suggest that TAK1 masked Y481 to interfere with Src access to TAB1.

### p38 enhances Src-mediated phosphorylation of both TAB1 and STAT1

**Figure 3:**
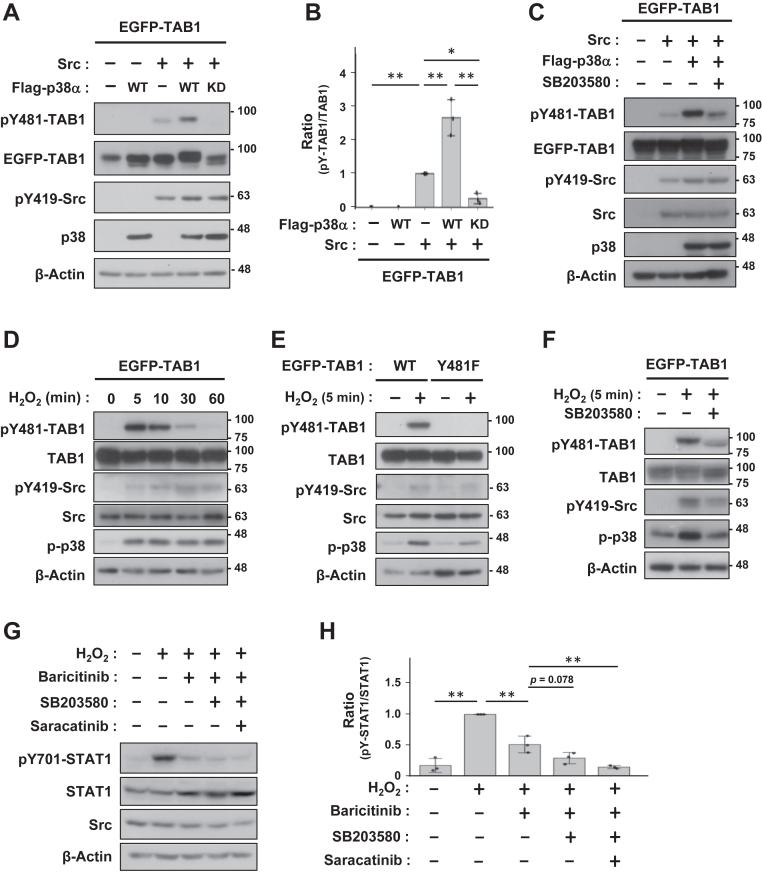
**p38 enhances Src-mediated TAB1-Y481 phosphorylation.***A* and *C*, COS-7 cells were transfected with EGFP-TAB1, Src, and the wild-type (WT) or kinase-dead (KD) mutant of Flag-p38α. SB203580 (10 μM) was added for 5 h (*C*). *B*, The relative quantification of pY481-TAB1, normalized to total TAB1, is presented as the mean ± SD of three independent experiments. *D*–*F*, COS-7 cells transfected with EGFP-TAB1 (WT or YF) were stimulated with 1 mM H_2_O_2_ for the indicated time. SB203580 was added as a pre-treatment for 5 h. *G*, COS-7 cells were pretreated with baricitinib (0.5 μM), SB203580 (10 μM), and saracatinib (0.5 μM) for 1 h and then treated with 1 mM H_2_O_2_ for 5 min. *H*, The relative quantification of pY701-STAT1, normalized to total-STAT1, is presented as the mean ± SD of three independent experiments. Cell lysates were immunoblotted with primary antibodies indicated (*A* and *C*–*G*). *p* values were calculated by one-way ANOVA followed by either Tukey’s HSD test was applied. ∗*p* < 0.05, ∗∗*p* < 0.01.

We examined the effects of p38 on pY481-TAB1 because p38 functionally cooperates with TAB1 as outlined above. The level of Src-induced pY481-TAB1 markedly increased when co-transfected with p38α (Fig. 3, *A* and *B*). This increase was not induced by the kinase-dead (KD) mutant of p38α (Fig. 3, *A* and *B*) and was suppressed by the p38 inhibitor SB203580 (Fig. 3*C*), indicating that p38 kinase activity was essential for this effect. A treatment with hydrogen peroxide, an oxidative stress condition known to activate both Src and p38, transiently induced pY481-TAB1 in a p38 activity-dependent manner (Fig. 3, *D*–*F*) (44). It is important to note that TAB1 was rapidly dephosphorylated despite the persistent activation of Src and p38.

Building on the well-established observation that STAT1 is activated by both JAK-dependent and JAK-independent pathways under oxidative stress (45, 46, 47), we focused on the latter—which may involve Src—and examined the contribution of p38. As expected, treatment with the JAK inhibitor baricitinib reduced H_2_O_2_-induced phosphorylation of endogenous STAT1 at Y701 by approximately half. We then assessed the roles of p38 and Src in the residual STAT1 activation that persisted in the presence of baricitinib. SB203580 showed a trend toward reducing the remaining STAT1 activation (*p* = 0.078), whereas the addition of the Src inhibitor saracatinib further suppressed it almost completely (Fig. 3, *I* and *J*). These results indicate that p38-mediated enhancement of Src activity influences not only TAB1 but also other Src substrates such as STAT1, thereby revealing a cellular/physiological response consistent with our proposed mechanism.

### p38-driven enhancement of pY481-TAB1 occurs without canonical TAB1-p38 interactions

**Figure 4:**
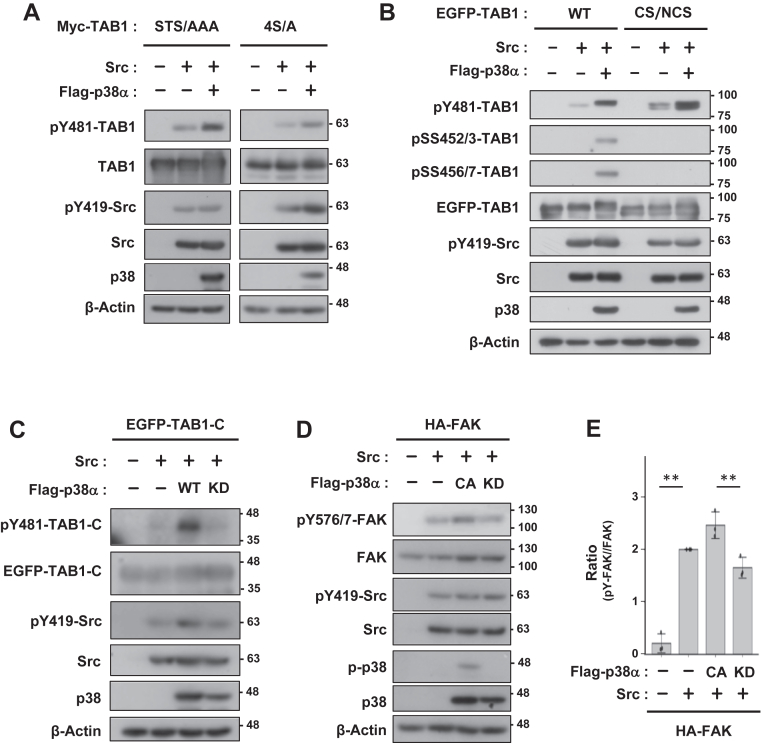
**The p38-mediated enhancement of pY481-TAB1 is independent of the serine/threonine phosphorylation of TAB1 by p38.***A*–*C*, COS-7 cells were transfected with Myc-tagged TAB1 (STS/AAA and 4S/A in a) or EGFP-tagged TAB1 (WT or CS/NCS in *B*), Src, and p38α. *D*, COS-7 cells were transfected with HA-tagged FAK, Src, and p38α. Cell lysates were immunoblotted with the primary antibodies indicated. *E*, the relative quantification of pY576/577-FAK, normalized to total FAK, is presented as the mean ± SD of three independent experiments. *p* values were calculated by one-way ANOVA followed by either Tukey’s HSD test was applied. ∗∗*p* < 0.01.

To clarify the mechanisms by which p38 promoted pY481-TAB1, we focused on the functional interactions between TAB1 and p38. To confirm the S/T phosphorylation of TAB1 by p38, we used the previously reported TAB1 STS/AAA (S423, T431, and S438 substituted to A) and 4SA (S452, S453, S456, and S457 substituted to A) mutants (48). TAB1 mutants still received elevated Src-mediated pY481-TAB1 in the presence of p38α (Fig. 4*A*). To investigate the role of the direct binding of TAB1 to p38, we used the TAB1 CS/NCS mutant (39), which has a lower binding ability to p38, resulting in a reduction in TAB1 S/T phosphorylation. The pY481 level of the TAB1-CS/NCS mutant was similar to that of wild-type TAB1 in the presence of p38α, even in TAB1 versions lacking the p38-mediated phosphorylation sites at S452/453 and pSS456/457 (Fig. 4*B*). This is consistent with the result showing that p38 increased pY481-TAB1 even in TAB1-C lacking the p38-binding region (Fig. 4*C*). These results confirmed that the p38-mediated enhancement of pY481-TAB1 did not require the previously reported p38-TAB1 interactions nor p38-dependent S/T phosphorylation. Based on the result showing that the phosphorylation of FAK at Y576, the known Src substrate with no functional interaction with p38, was also enhanced by CA p38α (Fig. 4, *D* and *E*), p38 may directly increase the tyrosine kinase activity of Src.

### p38 phosphorylates Src to increase Src kinase activity

**Figure 5:**
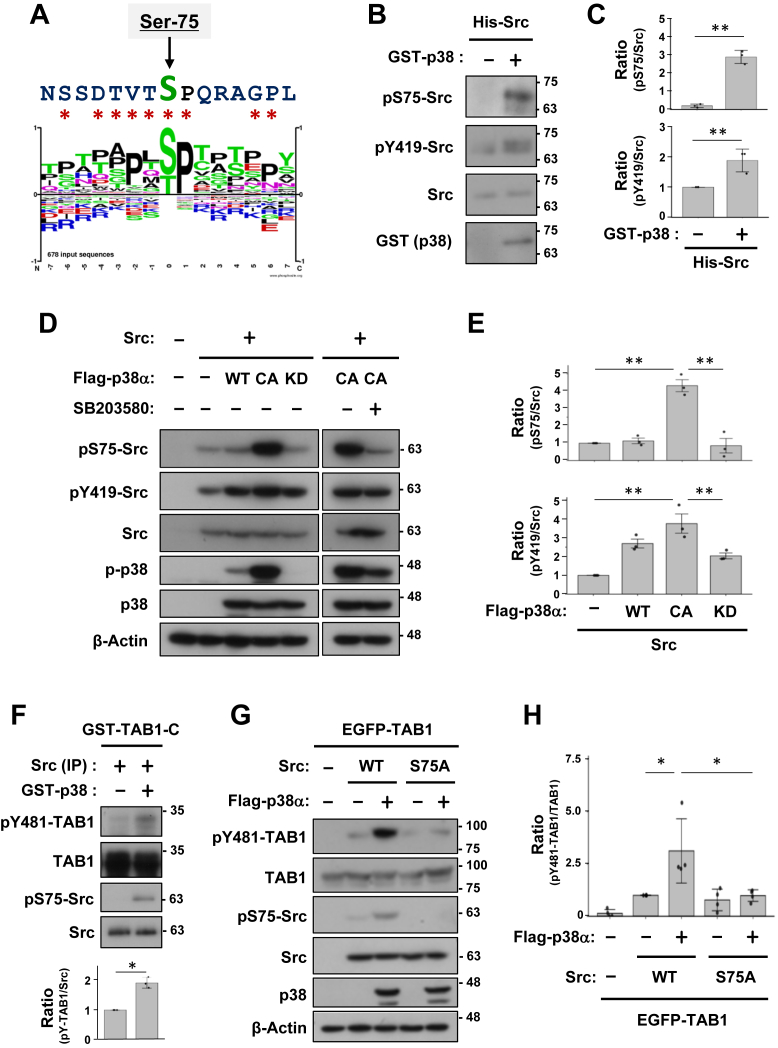
**p38 directly phosphorylates Src to increase its kinase activity.***A*, comparison between the substrate consensus sequence of p38α and the amino acid sequences of human Src around S75. *Asterisks* indicate amino acids that are common to both. *B*, an *in vitro* kinase assay using recombinant His-tagged Src and GST-tagged p38α proteins. Immunoblot analyses were performed with the primary antibodies indicated. *C*, the relative quantification of pS75-Src and pY419-Src, normalized to total Src, is presented as the mean ± SD of three independent experiments. *p* values were calculated by Welch’s two-tailed *t* test was applied. ∗∗*p* < 0.01. *D*, COS-7 cells were transfected with Src and p38α (WT, CA or KD), and then treated with SB203580 for 5 h. Immunoblot analyses were performed with the primary antibodies indicated. *E*, the relative quantification of pS75-Src and pY419-Src, normalized to total Src, is presented as the mean ± SD of three independent experiments. *p* values were calculated by one-way ANOVA followed by either Tukey’s HSD test was applied. ∗∗*p* < 0.01. *F*, Src immunoprecipitated from transfected COS-7 cells was preincubated with recombinant p38 for 60 min. After removal of p38, the tyrosine kinase activity of Src toward GST-TAB1-C at Y481 was evaluated by an additional 20-min incubation. The relative quantification of pY481-TAB1, normalized to total Src, is presented as the mean ± SD of three independent experiments. *p* values were calculated by Welch’s two-tailed *t* test was applied. ∗*p* < 0.05. *G*, COS-7 cells were transfected with EGFP-TAB1, Src (WT or S75A), and p38α. Immunoblot analyses were performed with the primary antibodies indicated. *H*, the relative quantification of pY481-TAB1, normalized to total TAB1, is presented as the mean ± SD of four independent experiments. *p* values were calculated by one-way ANOVA followed by either Tukey’s HSD test was applied. ∗*p* < 0.05.

We investigated whether p38 directly regulated Src activity, with a focus on S75 in the N-terminal region of Src. The sequence around S75 was very similar to the typical consensus sequence for the substrates of p38α, including a proline at the +1 position (Fig. 5*A*). Previous studies reported the phosphorylation of Src-S75 by some kinases other than p38; however, the function of this effect remains unclear (28, 29, 30, 31). To clarify the functional relationship between p38 and Src-S75 in the absence of TAB1, we conducted an *in vitro* kinase assay and found that recombinant p38 efficiently phosphorylates bacterially expressed Src at S75. This phosphorylation may, in turn, facilitate subsequent induction of Y419 phosphorylation (Fig. 5, *B* and *C*). The CA mutant of p38 significantly increased pS75-Src in COS-7 cells, and this effect was suppressed by the inhibition of p38 activity using SB203580 (Fig. 5*D*). In these experiments, co-expression of p38, including kinase-dead mutant, also increased the pY419 levels of Src (Fig. 5, *D* and *E*). Consistently, pretreatment of immunoprecipitated Src from transfected COS-7 cells with recombinant p38 *in vitro* significantly enhanced S75 phosphorylation and its tyrosine kinase activity toward recombinant GST-TAB1-C at Y481 (Fig. 5*F*). To investigate these regulatory dependencies further, the Src-S75A mutant was co-transfected with TAB1 and p38. The p38-mediated enhancement of pY481-TAB1 was reduced in the Src-S75A mutant (Fig. 5, *G* and *H*). However, pY419 of Src-S75A and pY481-TAB1 were still slightly increased by coexpressed p38, indicating the regulation of other residues besides S75 by p38 (see below). Collectively, these results indicate that p38 directly phosphorylated Src at S75 and allosterically increases the potency of its phosphoryl transfer ability to its substrates, including TAB1 and FAK.

### Differential patterns of substrate recognition by Src

Ample evidence suggests that the phosphorylation of Y419 and Y530 regulates Src activity. However, since some substrates are catalyzed independent of the phosphorylation state of Y419, this residue may also contribute to substrate recognition (25). In addition, the SH2 domain of Src, centered on R178 (corresponding to R175 in chicken Src), contributes to the formation of the closed inactive form by intramolecularly associating with pY530, but also contributes to the recognition of tyrosine-phosphorylated substrates by the active form with an open conformation (12, 14, 21, 22).

**Figure 6:**
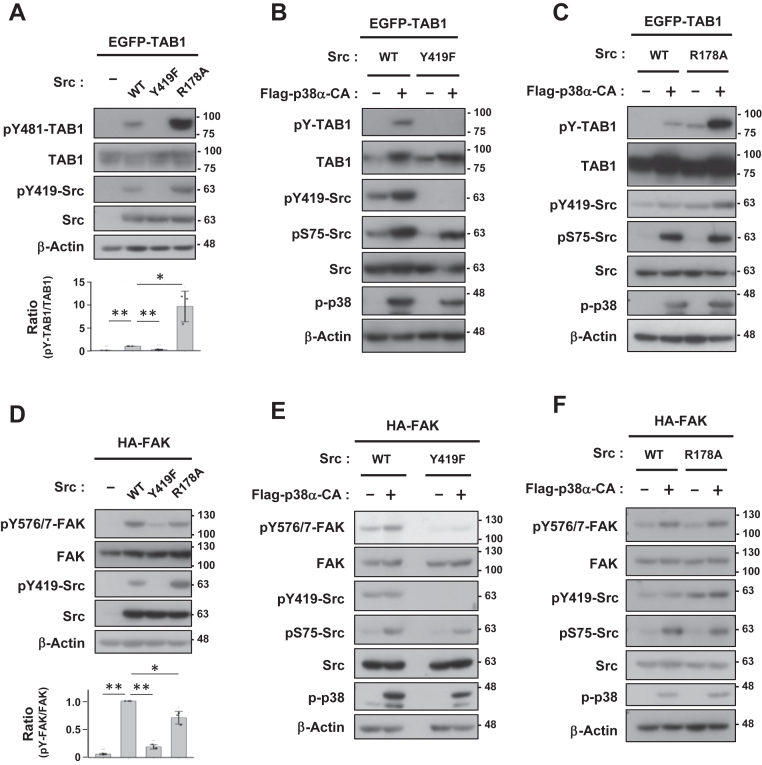
**Differences in TAB1 and FAK recognition patterns by Src**. *A*–*C*, COS-7 cells were transfected with EGFP-tagged TAB1 and Src (WT, Y419F and R178A) and active p38α-CA. *D*–*F*, COS-7 cells were transfected with HA-tagged FAK and Src (WT, Y419F and R178A) and active p38α-CA. Cell lysates were immunoblotted with primary antibodies as indicated. The relative quantification of pY481-TAB1/TAB1 and pY576/577-FAK/FAK, respectively, is presented as the mean ± SD of three independent experiments. *p* values were calculated by Dunnett’s multiple comparisons test was applied. ∗∗*p* < 0.01.

Based on this information, we generated Y419F and R178A mutants of Src to investigate the mechanisms by which Src recognizes the novel substrate TAB1 relative to those for FAK. TAB1-Y481 phosphorylation by the Src-Y419F mutant was completely undetectable (Fig. 6*A*). In contrast, the Src-R178A mutant maintained a high level of pY419, which coincided with the markedly higher phosphorylation of TAB1 (Fig. 6*A*). However, neither the co-expression of CA p38α nor an additional R178A mutation rescued the defective phosphorylation of TAB1-Y481 by Src-Y419F (Figs. 6*B* and S2), indicating that TAB1 phosphorylation in this setting was strictly dependent on Y419. Concerning the phosphorylation of TAB1 by Src, it was remarkable that this effect was even stronger by coexpression of the R178A mutant than by coexpression of p38α (Fig. 6*C*). In contrast, FAK was very weakly phosphorylated at Y576/577 by the Src-Y419F mutant and its phosphorylation by the R178A mutant was attenuated (Fig. 6*D*). The phosphorylation of FAK by Src was enhanced more by the coexpression of the R178A mutant than by the coexpression of p38α (Fig. 6*E*). In addition, no further induction by p38α was observed (Fig. 6*F*). These results show that the crosstalk of Src with TAB1 or FAK occurred through different mechanisms, with TAB1 being identified as the substrate that was reproducibly phosphorylated by Src in a pY419/pS75-dependent manner.

## Discussion

The main result of the present study is the identification of Y481 in the TAK1-binding domain of TAB1 as a new Src target site; this phosphorylation is strictly dependent on pY419-Src and is affected by intramolecular autoinhibition *via* the Src SH2 domain. Another important result is that p38 activated Src *via* direct non-canonical serine phosphorylation. Taken together with previous findings showing that TAB1 triggered p38 autoactivation and p38 induced TAB1 phosphorylation, complex signaling networks, such as positive/negative feedback loops, exist between these molecules. Since modifications to TAB1, particularly tyrosine phosphorylation, have not yet been examined in detail, the result showing a link to Src signaling will provide a new perspective on the physiological function of TAB1 independent of TAK1.

Important insights were obtained from the effects of TAK1, the main counterpart of TAB1. The crystal structure analysis showed that TAK1 directly interacted with TAB1 to mask Y481, which prevented substrate recognition by Src (Fig. 2*A*). pY481-TAB1 did not bind to TAK1 (Fig. 2*C*), indicating that Src only phosphorylated TAK1-unbound TAB1. These results also need to be considered in terms of the stoichiometric ratio of TAK1 to TAB1 in a cell, *i.e.*, whether inactive TAK1 is pre-bound to TAB1 and how much free TAB1 is present. In any case, a future focus of research needs to be new TAB1 functions independent of TAK1. The different phenotypes of TAK1 and TAB1 knockout mice clearly indicate the existence of TAK1-independent TAB1 functions (49, 50). The present study reveals a compelling feed-forward loop in which activated p38 triggers the non-canonical activation of Src, culminating in the phosphorylation of TAB1 at Y481. When considered alongside the mechanism of p38 autoactivation mediated by TAB1, this finding underscores a self-amplifying signaling axis that may play a pivotal role in sustaining or intensifying cellular responses. This loop may result in the sustained activation of p38 because it is not affected by the feedback inhibition of TAK1 *via* the p38-mediated serine/threonine phosphorylation of TAB1 (pST-TAB1), and may also affect the cytoplasm-nuclear shuttling of TAB1 because pST-TAB1 is known to be retained in the cytoplasm (48). Another possible partner of TAB1 is ligand-unbound EGFR (51). TAB1 stimulated with EGF dissociated from EGFR, which may involve the Y481 phosphorylation of TAB1 by Src activated by EGFR signaling.

Several SFKs, including Fyn, Lyn, and Hck, did not efficiently phosphorylate TAB1-Y481 (Figs. 1*F* and S1). However, since SFKs and TAK1 play important roles in lymphocyte activation (49, 52, 53, 54, 55), the function of TAB1 phosphorylation by SFKs in immune cells needs to be elucidated in more detail. On the other hand, S75 in the unique N-terminal domain of Src, a novel p38 target site for non-canonical activation, was also not conserved among SFKs (Fig. S3). These results indicate that the p38-Src-TAB1 pathway is specific to c-Src. The S75 phosphorylation of Src has been reported; for example, CDK1/CDC2-mediated S75 phosphorylation is known to activate Src during mitosis in HepG2 cells (28, 29, 56). Furthermore, p38 activity was required for the timely stable attachment of all kinetochores to spindle microtubules in the mitotic checkpoint (57). Collectively, these findings strongly suggest a role for non-canonical Src activation *via* p38 during mitosis. On the other hand, although not related to mitosis, S75 phosphorylation by CDK5 has been detected in human retinoblastoma cells (31). Similarly, CDK5-mediated S75 phosphorylation induced the ubiquitin-dependent degradation of Src to restrict the availability of active Src (30). While the present results offer new possibilities that differ from previous studies, the physiological function of the complex signaling network involving p38, Src, and TAB1 requires further investigation.

Consistent with previous findings, the Src-Y419F mutant phosphorylated FAK-Y576/577; however, the phosphorylation of TAB1-Y481 was strictly dependent on pY419 (Fig. 6, *A* and *B*). In contrast, the Src SH2 domain, particularly the R178 phosphotyrosine acceptor site, was shown to have a highly distinctive role in the phosphorylation of TAB1-Y481. The R178 mutation may induce a fully open conformation (12), thereby facilitating substrate recognition for TAB1-Y481. Conversely, the phosphorylation of FAK-Y576/577 was not significantly increased because the R178A mutation resulted in not only an open conformation, but also reduced binding affinity to the phospho-tyrosine residues of FAK (21, 22). These results suggest that FAK, owing to its dependence on the Src SH2 domain for recognition, is more appropriately considered an exception, whereas TAB1 likely represents a general Src substrate. It is important to note that the phosphorylation of TAB1 by Src-R178A without the SH2 function was further enhanced by the non-canonical phosphorylation of Src by p38, showing activation mechanisms independent of intramolecular repression by the SH2 domain. The S75 phosphorylation of Src in the unique domain may cause electrostatic repulsion between phosphorylated residues and negatively charged lipids on the membrane (58, 59, 60).

Although the involvement of known p38:TAB1 interaction interfaces has been ruled out, the possibility of an uncharacterized interface cannot be excluded. Furthermore, as described below, the presence of a novel phosphorylation site is also suggested. The PhoshoSitePlus database lists S306 as another phosphorylation site of Src, which also has a proline at position +1 and could be a substrate for p38. We showed that the S75A mutation reduced Src activity against TAB1 and FAK. S306 of the Src kinase domain is toward the protein surface, near the activation loop containing Y419 at about 15 Å. Besides, S306 is a component of the αC helix in the N-lobe of the Src kinase domain (19), which has been shown to be involved in affinity with ATP and protein substrates (61, 62, 63). Thus, phosphorylation of S306 by p38 can cause conformational changes in the helix, such as switching between αC-in and αC-out structures. These results suggest that S306 plays an important role in the activation of SFKs by p38, and more importantly in the case of Src, that S75 and S306 may cooperate to regulate its activity.

Although the present study found a unique signaling network, as described above, there are various limitations that need to be addressed. We demonstrated that hydrogen peroxide induced the phosphorylation of endogenous TAB1; however, its physiological function in the cell remains unknown. Furthermore, the types of cellular stimuli triggering the Src-TAB1 pathway and where phosphorylated TAB1 localizes in the cell and its functions have yet to be reported. These analyses will provide new directions for TAB1 and Src research.

In summary, we established a new direction for the Src signaling axis. Crosstalk between Src, p38, and TAB1, which function as key signaling molecules in the tumor microenvironment where various growth factors, cytokines, and oxidative stress cooperate, is attracting increasing interest. Since Src is widely activated in human cancers and its inhibitors are in clinical use, the phosphorylation of TAB1, for example, may be useful as a new biomarker of Src activation in tumors with chronic inflammation.

## Experimental procedures

### Cell culture

COS-7 and HCT116 cells were purchased from ATCC (American Type Culture Collection). COS-7 cells were cultured in Dulbecco’s Modified Eagle’s Medium (Nissui) containing 10% FBS, 4 mM L-glutamine, 100 U/ml penicillin, and 100 U/ml streptomycin (Meiji Seika Pharma) at 37 °C in 5% CO_2_. To establish the human c-Src-inducible HCT116 cell line, HCT116-c-Src, the pcDNA4/TO/c-Src plasmid (64) for the inducible expression of c-Src was transfected into HCT116/TR cells (65), which express the tetracycline repressor, by lipofection using Lipofectamine 2000 reagent (Invitrogen). Forty-eight hours after transfection, cells were selected in a growth medium containing 300 μg/ml geneticin and 300 μg/ml hygromycin for 2 to 3 weeks, and geneticin and hygromycin-resistant cell clones were isolated. The doxycycline-inducible expression of c-Src was assessed by Western blotting using an anti-Src monoclonal antibody (clone GD11, Millipore). HCT116-c-Src cells were cultured in Iscove’s Modified Dulbecco’s Medium IMDM (Wako) supplemented with 5% Tet-system approved FBS (Clontech), 100 U/ml penicillin, and 100 U/ml streptomycin.

### Antibodies and reagents

Phospho-specific monoclonal anti-TAB1 antibodies were generated using the rabbit immunospot array assay on a chip system, as previously described (41). The synthetic pY481-TAB1 peptide (pY-TAB1; EDGRVEP[pY]VDFAEFY), biotinylated pY-TAB1 peptide, and KLH-conjugated pY-TAB1 peptide were obtained from Eurofins (Tokyo, Japan). A rabbit was immunized with the KLH-conjugated pY-TAB1 peptide. IgG was purified, titrated by an enzyme-linked immunosorbent assay, and applied to Western blotting. Experiments using rabbits were approved by the Committee on Animal Experiments at the University of Toyama.

Primary antibodies for pY419-Src family kinase (2101S), total-Src (2110S), pT180/pY182-p38 (4511S), pY701-STAT1 (sc-136229), total-STAT1 (sc-346), pY576/577-FAK (3281S), and total-FAK (3285S) were purchased from Cell Signaling Technology. pS75-Src (ab79308) was from Abcam. Primary antibodies for p38α (sc-271120), TAB1 (sc-166138), phosphotyrosine (clone PY20, sc-508), GFP (sc-9996), and β-actin (sc-47778) were obtained from Santa Cruz Biotechnology. Dasatinib, saracatinib, SB203580, and doxycycline were obtained from Selleck; baricitinib was from MedChemExpress.

### Transfection of plasmid DNAs and siRNAs

The expression vectors for EGFP-tagged full-length, N-terminal, and C-terminal TAB1, Myc-tagged TAB1 Flag-tagged TAK1, the HA-tagged TAK1-TAB1 fusion protein, and Flag-tagged p38a were previously described (34, 42, 48, 66, 67). Src cDNA amplified from Huh7.5 cells was inserted into the pEF1-A expression vector (Invitrogen). Mutations were introduced by site-directed mutagenesis using KOD-Plus-Neo or KOD FX Neo (TOYOBO). Plasmid DNAs were transfected into COS-7 cells by Lipofectamine 3000 (Thermo Fischer Scientific) according to the manufacturer’s instructions. Twenty-four hours after transfection, cells were treated with regents for the indicated periods and then lysed by the following method. After the indicated procedures, cells were collected and used for Western blotting.

### Western blotting

Whole-cell lysates, prepared as previously described (68), were resolved using SDS-PAGE and transferred to an Immobilon-P nylon membrane (Merck KGaA). The membrane was treated with BlockAce (KAC Co., Ltd, Kyoto, Japan) and probed with the primary antibody at room temperature. Antibodies were detected using horseradish peroxidase-conjugated anti-rabbit, anti-goat, or anti-mouse IgG (DAKO) diluted in Can Get Signal solution (TOYOBO) or PBS containing 0.1% Tween 20 (Wako Pure Chemical Industries). Signals were detected with an enhanced chemiluminescence system (Thermo Fisher Scientific). Protein band intensities were quantified in ImageJ software using the Band/Peak quantification macro described previously (69). Phosphorylated protein levels were quantified relative to the corresponding total protein levels. The resulting normalized values, derived from three independent experiments (n = 3), were subjected to statistical analysis as outlined below.

### Immunoprecipitation

Cell lysates were diluted with an equal volume of dilution buffer (20 mM HEPES (pH 7.7), 2.5 mm MgCl_2_, 0.1 mM EDTA, 0.05% Triton X-100, 20 mM β-glycerophosphate, 1 mM sodium orthovanadate, 1 mM phenylmethylsulfonyl fluoride, 1 mM DTT, 10 μg/ml aprotinin, and 10 μg/ml leupeptin). After centrifugation, lysates were incubated with the anti-EGFP antibody at 4 °C overnight and then rotated with Dynabeads Protein G (Thermo Fisher Scientific) at 4 °C for 1.5 h. The beads were washed three times with wash buffer (1:1 mixture of whole-cell lysate buffer and dilution buffer).

### *In vitro* kinase assay

The recombinant human GST-TAB1-C protein (WT and Y481F) derived from *Escherichia coli* (34) was reacted with the recombinant human active GST-Src kinase derived from insect cells (Carna Bioscience) at 30 °C for 30 min in 30 μl of reaction buffer containing 20 mM HEPES (pH 7.6), 20 mM MgCl_2_, 0.2 mM ATP, 2 mM DTT, 20 mM β-glycerophosphate, and 0.1 mM sodium orthovanadate. Recombinant human 6 × His-Src protein derived from *E. coli* (RayBiotech, GA) was reacted with the recombinant human active GST-p38α kinase derived from *E. coli* (Carna Bioscience). After stopping the reaction by adding 30 μl of SDS–PAGE sample buffer, immunoblotting was performed as described above.

### Statistical analysis

All experiments were independently performed at least three times. Statistical analyses were conducted using R (version 4.4.2). For comparisons between two groups, Welch’s two-tailed *t* test was applied. For comparisons involving three or more groups, one-way ANOVA followed by either Tukey’s HSD test or Dunnett’s multiple comparisons test was used, as appropriate. Data are presented as mean ± SD with individual data points shown. Statistical significance is indicated as follows: *p* < 0.05 (∗), *p* < 0.01 (∗∗).

## Data availability

All data generated or analyzed during this study are included in this article and the supporting information file. Any additional data and original data presented in this article are available from the corresponding author upon reasonable request.

## Supporting information

This article contains supporting information.

## Conflicts of interest

The authors declare that they have no conflicts of interest with the contents of this article.

## References

[1] Simatou A., Simatos G., Goulielmaki M., Spandidos D.A., Baliou S., Zoumpourlis V. (2020). Historical retrospective of the SRC oncogene and new perspectives (Review). Mol. Clin. Oncol..

[2] Stehelin D., Varmus H.E., Bishop J.M., Vogt P.K. (1976). DNA related to the transforming gene(s) of avian sarcoma viruses is present in normal avian DNA. Nature.

[3] Brugge J.S., Erikson R.L. (1977). Identification of a transformation-specific antigen induced by an avian sarcoma virus. Nature.

[4] Collett M.S., Purchio A.F., Erikson R.L. (1980). Avian sarcoma virus-transforming protein, pp60src shows protein kinase activity specific for tyrosine. Nature.

[5] Turro E., Greene D., Wijgaerts A., Thys C., Lentaigne C., Bariana T.K. (2016). A dominant gain-of-function mutation in universal tyrosine kinase SRC causes thrombocytopenia, myelofibrosis, bleeding, and bone pathologies. Sci. Transl. Med..

[6] Irby R.B., Mao W., Coppola D., Kang J., Loubeau J.M., Trudeau W. (1999). Activating SRC mutation in a subset of advanced human colon cancers. Nat. Genet..

[7] Irby R.B., Yeatman T.J. (2000). Role of Src expression and activation in human cancer. Oncogene.

[8] Pelaz S.G., Tabernero A. (2022). Src: coordinating metabolism in cancer. Oncogene.

[9] Poh A.R., Ernst M. (2023). Functional roles of SRC signaling in pancreatic cancer: recent insights provide novel therapeutic opportunities. Oncogene.

[10] Okada M., Nada S., Yamanashi Y., Yamamoto T., Nakagawa H. (1991). CSK: a protein-tyrosine kinase involved in regulation of src family kinases. J. Biol. Chem..

[11] Xu W., Harrison S.C., Eck M.J. (1997). Three-dimensional structure of the tyrosine kinase c-Src. Nature.

[12] Bibbins K.B., Boeuf H., Varmus H.E. (1993). Binding of the Src SH2 domain to phosphopeptides is determined by residues in both the SH2 domain and the phosphopeptides. Mol. Cell Biol..

[13] Roussel R.R., Brodeur S.R., Shalloway D., Laudano A.P. (1991). Selective binding of activated pp60c-src by an immobilized synthetic phosphopeptide modeled on the carboxyl terminus of pp60c-src. Proc. Natl. Acad. Sci. U. S. A..

[14] Waksman G., Kominos D., Robertson S.C., Pant N., Baltimore D., Birge R.B. (1992). Crystal structure of the phosphotyrosine recognition domain SH2 of v-src complexed with tyrosine-phosphorylated peptides. Nature.

[15] Porter M., Schindler T., Kuriyan J., Miller W.T. (2000). Reciprocal regulation of hck activity by phosphorylation of Tyr527 and Tyr416: effect of introducing a high affinity intramolecular SH2 ligand. J. Biol. Chem..

[16] Meng Y., Roux B. (2014). Locking the active conformation of c-Src kinase through the phosphorylation of the activation loop. J. Mol. Biol..

[17] Kmiecik T.E., Shalloway D. (1987). Activation and suppression of pp60c-src transforming ability by mutation of its primary sites of tyrosine phosphorylation. Cell.

[18] Piwnica-Worms H., Saunders K.B., Roberts T.M., Smith A.E., Cheng S.H. (1987). Tyrosine phosphorylation regulates the biochemical and biological properties of pp60c-src. Cell.

[19] Pucheta-Martínez E., Saladino G., Morando M.A., Martinez-Torrecuadrada J., Lelli M., Sutto L. (2016). An allosteric cross-talk between the activation loop and the ATP binding site regulates the activation of src kinase. Sci. Rep..

[20] Thomas J.W., Ellis B., Boerner R.J., Knight W.B., White G.C., Schaller M.D. (1998). SH2- and SH3-mediated interactions between focal adhesion kinase and src. J. Biol. Chem..

[21] Eide B.L., Turck C.W., Escobedo J.A. (1995). Identification of Tyr-397 as the primary site of tyrosine phosphorylation and pp60src association in the focal adhesion kinase, pp125FAK. Mol. Cell Biol..

[22] Yeo M.G., Partridge M.A., Ezratty E.J., Shen Q., Gundersen G.G., Marcantonio E.E. (2006). Src SH2 arginine 175 is required for cell motility: specific focal adhesion kinase targeting and focal adhesion assembly function. Mol. Cell Biol..

[23] Wu J.C., Chen Y.C., Kuo C.T., Yu H.W., Chen Y.Q., Chiou A. (2015). Focal adhesion kinase-dependent focal adhesion recruitment of SH2 domains directs SRC into focal adhesions to regulate cell adhesion and migration. Sci Rep..

[24] Schaller M.D., Hildebrand J.D., Shannon J.D., Fox J.W., Vines R.R., Parsons J.T. (1994). Autophosphorylation of the focal adhesion kinase, ppl25FAK, directs SH2-Dependent binding of pp60src. Mol. Cell Biol..

[25] Cuesta-Hernández H.N., Contreras J., Soriano-Maldonado P., Sánchez-Wandelmer J., Yeung W., Martín-Hurtado A. (2023). An allosteric switch between the activation loop and a c-terminal palindromic phospho-motif controls c-Src function. Nat. Commun..

[26] Adan H., Guy S., Arulanandam R., Geletu M., Daniel J., Raptis L. (2022). Activated Src requires Cadherin-11, Rac, and gp130 for Stat3 activation and survival of mouse Balb/c3T3 fibroblasts. Cancer Gene Ther..

[27] Schreiner S.J., Schiavone A.P., Smithgall T.E. (2002). Activation of STAT3 by the src family kinase Hck requires a functional SH3 domain. J. Biol. Chem..

[28] Stover D.R., Liebetanz J., Lydon N.B. (1994). Cdc2-mediated modulation of pp60c-src activity. J. Biol. Chem..

[29] Shenoy S., Chackalaparampil I., Bagrodia S., Lin P.H., Shalloway D. (1992). Role of p34cdc2-mediated phosphorylations in two-step activation of pp60c-src during mitosis. Proc. Natl. Acad. Sci. U. S. A..

[30] Pan Q., Qiao F., Gao C., Norman B., Optican L., Zelenka P.S. (2011). Cdk5 targets active Src for ubiquitin-dependent degradation by phosphorylating Src(S75). Cell Mol. Life Sci..

[31] Kato G., Maeda S. (1999). Neuron-specific Cdk5 kinase is responsible for mitosis-independent phosphorylation of c-Src at Ser75 in human Y79 retinoblastoma cells. J. Biochem..

[32] Sakurai H. (2012). Targeting of TAK1 in inflammatory disorders and cancer. Trends Pharmacol. Sci..

[33] Ono K., Ohtomo T., Sato S., Sugamata Y., Suzuki M., Hisamoto N. (2001). An evolutionarily conserved motif in the TAB1 C-terminal region is necessary for interaction with and activation of TAK1 MAPKKK. J. Biol. Chem..

[34] Sakurai H., Miyoshi H., Mizukami J., Sugita T. (2000). Phosphorylation-dependent activation of TAK1 mitogen-activated protein kinase kinase kinase by TAB1. FEBS Lett..

[35] Cheung P.C.F., Campbell D.G., Nebreda A.R., Cohen P. (2003). Feedback control of the protein kinase TAK1 by SAPK2a/p38alpha. EMBO J..

[36] Shin M.S., Shinghirunnusorn P., Sugishima Y., Nishimura M., Suzuki S., Koizumi K. (2009). Cross interference with TNF-alpha-induced TAK1 activation *via* EGFR-mediated p38 phosphorylation of TAK1-binding protein 1. Biochim. Biophys. Acta.

[37] Ge B., Gram H., Di Padova F., Huang B., New L., Ulevitch R.J. (2002). MAPKK-independent activation of p38alpha mediated by TAB1-dependent autophosphorylation of p38alpha. Science.

[38] De Nicola G.F., Martin E.D., Chaikuad A., Bassi R., Clark J., Martino L. (2013). Mechanism and consequence of the autoactivation of p38α mitogen-activated protein kinase promoted by TAB1. Nat. Struct. Mol. Biol..

[39] De Nicola G.F., Bassi R., Nichols C., Fernandez-Caggiano M., Golforoush P.A., Thapa D. (2018). The TAB1-p38α complex aggravates myocardial injury and can be targeted by small molecules. JCI Insight.

[40] Hornbeck P.V., Kornhauser J.M., Tkachev S., Zhang B., Skrzypek E., Murray B. (2012). PhosphoSitePlus: a comprehensive resource for investigating the structure and function of experimentally determined post-translational modifications in man and mouse. Nucleic Acids Res..

[41] Ozawa T., Piao X., Kobayashi E., Zhou Y., Sakurai H., Andoh T. (2012). A novel rabbit immunospot array assay on a chip allows for the rapid generation of rabbit monoclonal antibodies with high affinity. PLoS One.

[42] Sakurai H., Nishi A., Sato N., Mizukami J., Miyoshi H., Sugita T. (2002). TAK1-TAB1 fusion protein: a novel constitutively active mitogen-activated protein kinase kinase kinase that stimulates AP-1 and NF-κB signaling pathways. Biochem. Biophys. Res. Commun..

[43] Brown K., Vial S.C.M., Dedi N., Long J.M., Dunster N.J., Cheetham G.M.T. (2005). Structural basis for the interaction of TAK1 kinase with its activating protein TAB1. J. Mol. Biol..

[44] Aikawa R., Komuro I., Yamazaki T., Zou Y., Kudoh S., Tanaka M. (1997). Oxidative stress activates extracellular signal-regulated kinases through Src and Ras in cultured cardiac myocytes of neonatal rats. J. Clin. Invest..

[45] Simon A.R., Rai U., Fanburg B.L., Cochran B.H. (1998). Activation of the JAK-STAT pathway by reactive oxygen species. Am. J. Physiol. Cell Physiol..

[46] Rane S.G., Reddy E.P. (2002). JAKs, STATs and Src kinases in hematopoiesis. Oncogene.

[47] Silva C.M. (2004). Role of STATs as downstream signal transducers in Src family kinase-mediated tumorigenesis. Oncogene.

[48] Wolf A., Beuerlein K., Eckart C., Weiser H., Dickkopf B., Müller H. (2011). Identification and functional characterization of novel phosphorylation sites in TAK1-binding protein (TAB) 1. PLoS One.

[49] Sato S., Sanjo H., Takeda K., Ninomiya-Tsuji J., Yamamoto M., Kawai T. (2005). Essential function for the kinase TAK1 in innate and adaptive immune responses. Nat. Immunol..

[50] Komatsu Y., Shibuya H., Takeda N., Ninomiya-Tsuji J., Yasui T., Miyado K. (2002). Targeted disruption of the Tab1 gene causes embryonic lethality and defects in cardiovascular and lung morphogenesis. Mech. Dev..

[51] Guo G., Gong K., Beckley N., Zhang Y., Yang X., Chkheidze R. (2022). EGFR ligand shifts the role of EGFR from oncogene to tumour suppressor in EGFR-amplified glioblastoma by suppressing invasion through BIN3 upregulation. Nat. Cell Biol..

[52] Sun L., Deng L., Ea C.K., Xia Z.P., Chen Z.J. (2004). The TRAF6 ubiquitin ligase and TAK1 kinase mediate IKK activation by BCL10 and MALT1 in T lymphocytes. Mol. Cell.

[53] Shinohara H., Yasuda T., Aiba Y., Sanjo H., Hamadate M., Watarai H. (2005). PKC beta regulates BCR-mediated IKK activation by facilitating the interaction between TAK1 and CARMA1. J. Exp. Med..

[54] Palacios E.H., Weiss A. (2004). Function of the Src-family kinases, Lck and Fyn, in T-cell development and activation. Oncogene.

[55] Salmond R.J., Filby A., Qureshi I., Caserta S., Zamoyska R. (2009). T-cell receptor proximal signaling *via* the Src-family kinases, Lck and Fyn, influences T-cell activation, differentiation, and tolerance. Immunol. Rev..

[56] Kato G., Maeda S. (1995). Novel phosphorylation at a mitotic site, serine 75, in human pp60c-src from unsynchronized human tumor cells having a spherical morphology. Biochem. Biophys. Res. Commun..

[57] Lee K., Kenny A.E., Rieder C.L. (2010). P38 mitogen-activated protein kinase activity is required during mitosis for timely satisfaction of the mitotic checkpoint but not for the fidelity of chromosome segregation. Mol. Biol. Cell.

[58] Pérez Y., Gairí M., Pons M., Bernadó P. (2009). Structural characterization of the natively unfolded N-Terminal domain of human c-Src kinase: insights into the role of phosphorylation of the unique domain. J. Mol. Biol..

[59] Pérez Y., Maffei M., Igea A., Amata I., Gairí M., Nebreda A.R. (2013). Lipid binding by the Unique and SH3 domains of c-Src suggests a new regulatory mechanism. Sci Rep.

[60] Aponte E., Lafitte M., Sirvent A., Simon V., Barbery M., Fourgous E. (2022). Regulation of Src tumor activity by its N-terminal intrinsically disordered region. Oncogene.

[61] Sicheri F., Moarefi I., Kuriyan J. (1997). Crystal structure of the Src family tyrosine kinase Hck. Nature.

[62] Foda Z.H., Shan Y., Kim E.T., Shaw D.E., Seeliger M.A. (2015). A dynamically coupled allosteric network underlies binding cooperativity in Src kinase. Nat. Commun..

[63] Huang H., Zhao R., Dickson B.M., Skeel R.D., Post C.B. (2012). αC helix as a switch in the conformational transition of Src/CDK-like kinase domains. J. Phys. Chem. B.

[64] Nakayama Y., Matsui Y., Takeda Y., Okamoto M., Abe K., Fukumoto Y. (2012). c-Src but not fyn promotes proper spindle orientation in early prometaphase. J. Biol. Chem..

[65] Soeda S., Nakayama Y., Honda T., Aoki A., Tamura N., Abe K. (2013). v-Src causes delocalization of Mklp1, Aurora B, and INCENP from the spindle midzone during cytokinesis failure. Exp. Cell Res..

[66] Zhou Y., Oki R., Tanaka A., Song L., Takashima A., Hamada N. (2023). Cellular stress induces non-canonical activation of the receptor tyrosine kinase EphA2 through the p38-MK2-RSK signaling pathway. J. Biol. Chem..

[67] Maeno K., Sada K., Kyo S., Miah S.M.S., Kawauchi-Kamata K., Qu X. (2003). Adaptor protein 3BP2 is a potential ligand of src homology 2 and 3 domains of lyn protein-tyrosine kinase. J. Biol. Chem..

[68] Sakurai H., Miyoshi H., Toriumi W., Sugita T. (1999). Functional interactions of transforming growth factor β-activated kinase 1 with IκB kinases to stimulate NF-κB activation. J. Biol. Chem..

[69] Ohgane K., Yoshioka H. (2019).

