# Supplementary material for: The stress-activated kinase p38 mediates non-canonical activation of Src and tyrosine phosphorylation of the adapter protein TAB1
Source: J Biol Chem. 2026 Jan 23;302(3):111200. doi: 10.1016/j.jbc.2026.111200 (PMC12925544; doi:10.1016/j.jbc.2026.111200)
Supplement: Supplementary Figures [file mmc1.pdf]

Supplementary Figure S1

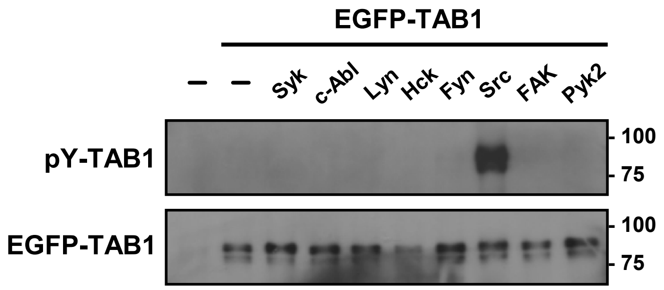

Supplementary Figure S1. Src selectively phosphorylates TAB1.

COS-7 cells were transfected with EGFP-tagged TAB1 and several tyrosine kinases as indicated. Cell lysates and immunoprecipitates with an anti-GFP antibody were immunoblotted with anti-phosphotyrosine and anti-GFP antibodies.

Supplementary Figure S2

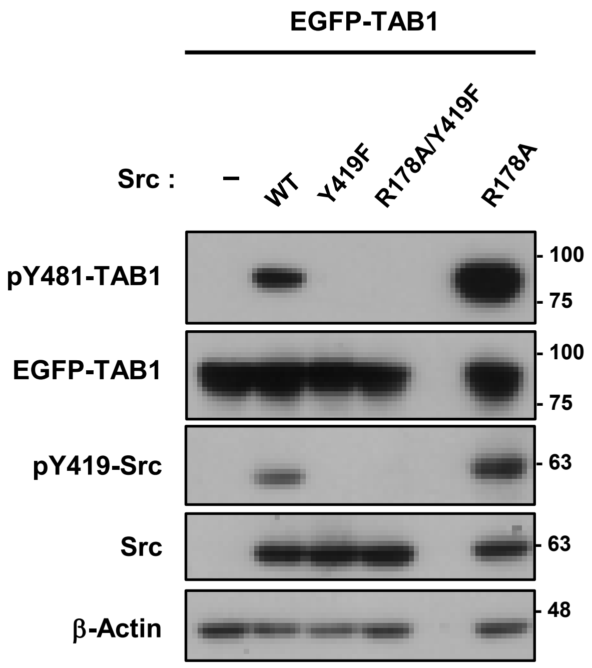

Supplementary Figure S2. Effect of R178A mutation on Src-Y419F activity.
COS-7 cells were transfected with EGFP-tagged TAB1 and Src (WT, Y419F, Y419/R178A and R178A). Cell lysates were immunoblotted with the primary antibodies indicated.

Supplementary Figure S3

```
sp|P12931|SRC_HUMAN/50-108  PSAAFAPA-------AAEPKLFGGFNSSDTVTSPQRAGPLAGGVTTFVALYDYESRTETDLSFKKG
sp|P09769|FGR_HUMAN/47-101  -FAHIPNYSNFSSQA-----INPGFL-----DSGTIRGVSGIGVTLFIALYDYEARTEDDLTFTKG
sp|P08631|HCK_HUMAN/63-102  PNS------------HNSNTPGIR--------------EAGSEDIIVVALYDYEAIHHEDLSFQKG
sp|P07947|YES_HUMAN/50-115  TAVNFSSLSMTPFGGSSGVTPFGGASSSFSVVPSSYPAGLTGGVTIFVALYDYEARTTEDLSFKKG
sp|P07948|LYN_HUMAN/44-87   PVP------------ESQLLPGQRF------QTK----DPEEQGDIVVALYPYDGIHPDDLSFKKG
sp|P06241|FYN_HUMAN/44-106  V-TSIPNYNNFHAAGGQGLTVFGGVNSSSHTGTLRT--RGGTGVTLFVALYDYEARTEDDLSFHKG
sp|P06239|LCK_HUMAN/42-85   SEV------------RDPLVTYEGS------NPP----ASPLQDNLVIALHSYEPSHDGDLGFEKG
sp|P51451|BLK_HUMAN/35-82   APP------------LPPLVVFNHL------TPPPPDEHLDEDKHFVVALYDYTAMNDRDLQMLKG
```

Supplementary Figure S3. Alignment of amino acid sequences around Src S75 in SFKs.
S75 in the unique N-terminal domain of human Src, a novel p38 target site for non-canonical activation, was also not conserved among human SFKs.
